# Congenital Infection Influence on Early Brain Development Through the Gut-Brain Axis

**DOI:** 10.3389/fnins.2022.894955

**Published:** 2022-06-30

**Authors:** Gregory W. Kirschen, Snigdha Panda, Irina Burd

**Affiliations:** ^1^Department of Gynecology and Obstetrics, The Johns Hopkins Hospital, Baltimore, MD, United States; ^2^Integrated Center for Fetal Medicine, The Johns Hopkins Hospital, Baltimore, MD, United States; ^3^Department of Biology, Johns Hopkins University, Baltimore, MD, United States

**Keywords:** microbiome, virome, TORCH infections, neuroinflammation, neurodevelopmental disorders

## Abstract

The mechanisms by which various pathogens cause congenital infections have been studied extensively, aiding in the understanding of the detrimental effects these infections can have on fetal/neonatal neurological development. Recent studies have focused on the gut-brain axis as pivotal in neurodevelopment, with congenital infections causing substantial disruptions. There remains controversy surrounding the purported sterility of the placenta as well as concerns regarding the effects of exposure to antibiotics used during pregnancy on neonatal microbiome development and how early exposure to microbes or antibiotics can shape the gut-brain axis. Long-term neurodevelopmental consequences, such as autism spectrum disorder, attention deficit hyperactivity disorder, and cerebral palsy, may be attributable, in part, to early life infection and changes in the immature gut microbiome. The goal of this review is thus to critically evaluate the current evidence related to early life infection affecting neurodevelopment through the gut-brain axis.

## Introduction

Congenital infections are a group of diseases that are present from the fetal into the early postnatal stage of life, which have been found to have adverse effects on metabolic, microbiome, and neurological development in the neonate (Surtees et al., 1990; Mechtler and Kinkel, 1993; Terrazzan Nutricionist et al., 2020). TORCH is a common acronym used to refer to a set of these infections: (T)oxoplasmosis, (O)ther agents such as syphilis and parvovirus B19, Rubella, (C)ytomegalovirus infection (CMV), and (H)erpes simplex virus (HSV) (Macedo-da-Silva et al., 2020; Jaan and Rajnik, 2022). In addition, recent studies have been conducted to understand the effects of other viruses that cause congenital infection—such as Zika virus (ZIKV) and the novel SARS-CoV-2 coronavirus (virus causing COVID-19)—on fetal development (Hoffman et al., 2021; Nunez et al., 2021). While the pathophysiology of the congenital infections has been investigated at length, recent developments in metabolomics and cellular physiology have begun to elucidate metabolic adaptations and derangements in the fetus that accompany these infections.

Another form of fetal infection, which usually occurs around the time of delivery or in the setting of premature, pre-labor rupture of membranes (PPROM) is called chorioamnionitis, also known as intraamniotic infection or inflammation (III) (Cappelletti et al., 2020). Bacteria ascend the female genital tract and infect the membranes surrounding the fetus as well as amniotic fluid, resulting in a pro-inflammatory state in the fetus (Kemp, 2014). III is a risk factor for adverse long-term neurodevelopmental outcomes including cerebral palsy, although the mechanisms leading to neurocognitive dysfunction remain incompletely understood (Huleihel et al., 2004; Elovitz et al., 2011; Brown et al., 2017).

Recently, the role of the gut-brain axis has come to light as affecting myriad functions important for normal neonatal health and development, including cognitive development (Griffiths and Mazmanian, 2018). Furthermore, although not frequently thought about in clinical practice, antibiotic exposure *in utero* likely has effects on neonatal microbiome development, which in turn could influence brain development (Grech et al., 2021). There is controversy surrounding the sterility vs. colonization of the placenta and fetus, with the classic teaching that the fetal gut and placenta are sterile, but some emerging evidence refuting this (Aagaard et al., 2014; Briana et al., 2021; Kennedy et al., 2021). Such colonization could have far-reaching implications for fetal development, as the developing gut microbiome has been found to be important for the manifestation of chronic diseases including obesity, metabolic dysfunction, diabetes in addition to neurological disorders such as autism spectrum disorder (ASD) and multiple sclerosis (MS) (Tilg and Kaser, 2011; Barlow et al., 2015; Cryan et al., 2020). In this review article, we focus on development of the gut-brain axis in the fetus and neonate, with attention to how these become disrupted in the setting of congenital infection/inflammation or exposure to antibiotics. In the following sections, we critically appraise the influence of different case examples of infectious diseases on fetal gut-brain development.

## Microglial Activation and Inflammation in the Neonatal Gut-Brain Axis

Microglia, the resident immune cells of the brain, derive from erythromyeloid progenitors in the extraembryonic yolk sac and establish their place in the fetal brain early in development, prior to neurogenesis or blood brain barrier (BBB) formation in human (Kracht et al., 2020). They are important not only for immune function and response in the brain, but also for synaptic pruning and modulating neurogenesis based on experiments in rodents (Sierra et al., 2010; Abdel-Haq et al., 2019). A genetic fate mapping study in rodents identified microglia as a highly dynamic, self-renewing population, and in the absence of systemic or local inflammation compromising BBB function, remain separate from peripheral bone marrow-derived monocyte/macrophage populations (Tay et al., 2017). Human microglia gain immune surveillance and phagocytic function early in fetal life (from 9 to 18 weeks of gestation) (Kracht et al., 2020).

**FIGURE 1:**
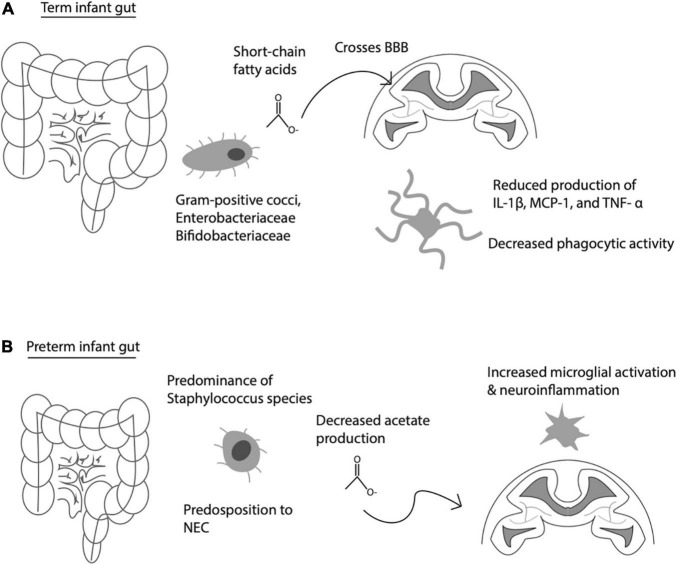
Communication between gut-derived short chain fatty acids (SCFA) and brain immune activity. **(A)** Normal interaction between gut-derived SCFA, which cross the blood brain barrier (BBB) to influence microglia activity under physiological conditions. **(B)** Premature infant gut with altered gut flora, predisposition to necrotizing enterocolitis (NEC), decreased acetate production, leading to increased microglial activation, and increased risk of neuroinflammation.

The gut-brain axis actively shapes microglial behavior and development. Globally, the gut microbiome influences the development of the host’s innate and adaptive immune responses (Abdel-Haq et al., 2019). This holds not only for the host’s peripheral immune cells, but also centrally within the brain. For instance, Erny et al. (2015) discovered that the host microbiome contributes greatly to microglial homeostasis. Germ-free mice exhibit severe impairments in microglial function, for instance defects in genetic regulation of immune activation and signal transduction via the interferon and Jak3/STAT1 pathway (Erny et al., 2015). It is likely that these effects occur via alterations in short-chain fatty acid (SCFA) signaling, as gut bacteria are key metabolizers of nutrition-derived fatty acids important for global and central nervous system (CNS)-specific immune function, as restoration of SCFA in germ-free mice rescued microglial function (Erny et al., 2015; Venegas et al., 2019; Figure 1).

When infection is present during pregnancy, inflammatory products—such as those associated with congenital infections and III—trigger microglial activation, resulting in neuroinflammation marked by the presence of IL-1β, TNF-α, and IL-6 (Lu and Claud, 2019). Microglial activation is also evidenced by white matter damage, which was found in animal studies modeling maternal infection utilizing LPS-induced mice to understand the role of microbiota inflammation on neurodevelopment (Burd et al., 2010; Elovitz et al., 2011). These same animal models have also linked the regulation of neural activity to the microbiome’s activation of microglia, further providing direct evidence of a reciprocally communicating gut-brain axis (Cryan et al., 2020). Antibiotic treatment in pregnant women can influence gut microbiome composition, specifically preterm neonates exposed to antibiotics demonstrating fewer *Frimicutes* and more *Proteobacteria*, accompanied by altered short chain fatty acid (SCFA) profiles, vs. those not exposed (Dahl et al., 2018). In turn, SCFAs produced by gut bacteria can cross the blood brain barrier and alter microglia cytokine release and dampen phagocytic activity *in vitro* (see Figure 1; Wenzel et al., 2020). While term infants have predominantly gram positive cocci, *Enterobacteriaceae* and *Bifidobacteriaceae* species, preterm infants have altered speciation, including higher abundance of *Strephyloccaceae* and delayed *Bifidobacteriaceae* colonization, associated with lower acetate levels, which may in turn lead to higher levels of microglial activation and neuroinflammation among preterm infants (Tauchi et al., 2019; Figure 1). This is corroborated by the finding of increased numbers of activated microglia in the brains of the preterm human brain, especially in the developing white matter (Supramaniam et al., 2013). These findings highlight the potential influence of the microbiota have on neuro-immune development.

It is also important to mention the role of the vagus nerve at the interface of the gut and brain interaction. Vagal nerve afferents innervate the gastrointestinal tract along its length, most notably in the form of the myenteric plexus, a network of neurons and glia between the smoothe and longitudinal muscle layers of the intestinal wall (Phillips and Powley, 2007). Testing the effects of the vagus nerve on the connection between brain and gut has historically been achieved by performing vagotomy, the surgical severing of the vagus nerve. A well-known phenomenon related to inflammation in general is the associated anorexia or lack of appetite. In mice, the mechanism underlying this effect has been experimentally determined to require vagal innervation. For instance, mice treated with lipopolysaccharide (LPS), a potent pro-inflammatory mediator, exhibit decreased food-motivated behavior, an effect that is inhibited by subdiaphragmatic vagotomy (8,590,821). Vagotomy does not, however, inhibit the body’s pyogenic response to proinflammatory cytokines, suggesting that the vagus nerve is responsible for some, but not all of the physiological responses to pathogens (Luheshi et al., 2000). Furthermore, SCFAs, produced by gut bacterial fermentation and known to cross the blood brain barrier (BBB), have been shown to improve survival in a mouse model of sepsis, an effect that is actually inhibited by surgical vagotomy, suggesting the vagus nerve’s importance in mediating communication between gut and brain in the context of severe infection (Okumura et al., 2021). Apart from the vagus nerve’s effect on food intake and food-related behavior during infection, it has also been hypothesized that diseases affecting both gut and brain may stem from abnormal connectivity or pathology involving the vagus nerve. For instance, Parkinson disease, which involves not only the well-known neurological signs and symptoms of bradykinesia, resting tremor, muscle rigidity, and postural instability, is accompanied by changes in function of the gastrointestinal tract, including colonic dysmotility and constipation. It has been posited that alpha-synuclein, a pathological misfolded protein implicated in Parkinson disease pathogenesis, actually spreads via the vagus nerve, potentially explaining an underlying common pathogenesis involving the CNS and GI tract (Breen et al., 2019).

In the following sections, we first explore the roles of congenitally acquired viral pathogens in triggering abnormal communication between the brain and gut.

## RNA Virus Infections’ Influence on Gut-Brain Development: Focus on Emerging Pathogens

We will examine how several case examples of RNA virus infections shape the fetal/neonatal gastrointestinal and neurological systems and attempt to draw connections between the two. The emergence of novel infections such as (Zika virus) ZIKV and COVID-19 has sparked investigation to understand the extent to which they alter the fetal microbiome, and thus, fetal neurodevelopment through the gut-brain axis. We group these two viruses together in this section as they share several commonalities, including that they are both positive-sense RNA viruses that have potential for vertical transmission (more common with ZIKV), and have demonstrated adverse perinatal and neonatal outcomes and characteristic placental pathologies in infected individuals, and limited therapeutic options at present (McBroom, 2021).

It has been established that congenital ZIKV has significant impacts on fetal brain development, resulting in microcephaly in 0.15–2% of cases, neural tube defects (0.1% of cases), ocular abnormalities (0.3% of cases), and high levels of proinflammatory mediators (Coelho and Crovella, 2017; Cragan et al., 2017; Lum et al., 2017; Nguyen et al., 2017). We have previously shown that ZIKV inflicts neuroinflammation by evoking an IL-1β response in the placenta (Lei et al., 2019). Current studies relating ZIKV to the gut microbiome focus largely on adult rodent models. For instance, non-pregnant adult mice infected with ZIKV exhibit significant decrease in bacteria belonging to the *Actinobacteria* and *Firmicutes* phyla while there was an increase in *Deferribacteres* and *Spirochaetes* phyla when compared to non-infected mice, as determined by next-generation sequencing (Correa et al., 2021). What impact does this ZIKV-induced gut dysbiosis have on the developing fetus? Although direct evidence is sparse for how ZIKV may alter the host gut microbiome to influence brain development, this may occur via changes in host immune response. For instance, ZIKV-infected mice depleted of *Stat2* exhibit more severe infection, as measured by survival, mobility/paralysis, and body weight as compared to *Stat2*-intact mice, suggesting a role of the interferon-STAT2 pathway in ZIKV pathogenesis (Tripathi et al., 2017). Alterations in gut flora including *Salmonella* infection and abundance of butyrate-producing organisms can lead to changes in STAT2-signaling, suggesting one pathway by which ZIKV may exert its pathogenesis (Wilson et al., 2019; Chemudupati et al., 2020).

Aside from direct effects of ZIKV on the developing gut and brain, there may be underlying genetic susceptibility to ZIKV-related congenital infection. For instance, Santos et al. (2019) found that a single nucleotide polymorphism (SNP) in the Toll-like receptor 3 (TLR3) exhibited increased odds of congenital ZIKV infection vs. controls lacking this SNP. The investigators further found that a SNP in the tumor necrosis factor alpha (TNF-α) gene conferred a heightened risk of microcephaly compared to those babies lacking this SNP. Likewise, alterations in epigenetic programming may influence the natural course of ZIKV infection. Decreased methylation of plasminogen activator inhibitor-1 (PAI-1, a regulator of innate immunity) was associated with a higher risk of microcephaly, for example (Nwanaji-Enwerem et al., 2021). These results suggest that genetic and epigenetic regulatory changes related to inflammatory cascades and immune activation may predispose to ZIKV contraction and more severe disease.

Due to the evolving and novel nature of the COVID-19 infection and the ongoing studies associated with it, a comprehensive understanding of its effect on the development of the fetal microbiome and neurological development has yet to be exhaustively investigated. Thus far, COVID-19 has been associated with adverse perinatal outcomes including increased risk of preterm birth, intrauterine growth restriction, and stillborn (McBroom, 2021; Dubucs et al., 2022). One review explored the emerging pediatric inflammatory syndrome accompanying COVID-19 infections in children, citing differences in the pediatric gut microbiome as responsible for the varying responses to infection (Mondal et al., 2021). The investigators analyzed stool samples 30 days after resolution of COVID-19 infection and found that there was a significant lower representation of gut microbiota in infected children compared to the healthy controls (Mondal et al., 2021; Yeoh et al., 2021). This is indicative of the infection eliciting an inflammatory response that ultimately altered the pediatric microbiome. These alterations in gut microflora may be associated with the increasingly reported neuropsychiatric phenomena that have been associated with COVID-19 infection, although this remains to be definitively demonstrated especially in the pediatric population (Mondal et al., 2021). Do genetic or epigenetic differences in the population predispose to congenital COVID acquisition and severity of infection? It has been reported that polymorphisms in the *ABO* gene as well as genes related to viral entry (e.g., angiotensin-converting enzyme 2, *ACE2*) have been associated with increased propensity to developing COVID-19 infection in adults, although these findings remain to be replicated in the setting of congenital infections (Benetti et al., 2020; Vargas-Alarcon et al., 2022). Epigenetic factors that have been associated with COVID-19 infection include methylation of the gene encoding *ACE2*, which also interestingly differs by sex, with females showing less DNA methylation as compared to males (Cardenas et al., 2021). Again, whether this translates into greater transmissibility across the placenta or confers more severe fetal infection remains to be seen.

Based on the above emerging evidence, it seems plausible that ZIKV and COVID-19 may lead to alterations in perinatal and early life gut-brain interactions. Longitudinal studies following those infected will be required to draw definitive conclusions regarding long-term effects on the gut microbiome and health outcomes across the lifespan.

## DNA Virus Infections’ Influence on Gut-Brain Development

We next focus on several DNA viruses that have also been implicated in development of neural-gut interactions. Parvovirus B19 is a single-stranded DNA virus that can cross the human placenta and lead to severe fetal anemia and hydrops fetalis via inhibition of fetal erythropoiesis (de Jong et al., 2006). Experimental studies in animals have shown that parvovirus not only affects the hematopoietic system, but also the developing gut. Parvovirus-infected Wister rats exhibit intestinal dysbiosis, with reduction in probiotic bacteria of *Parabacteroides* and *Butyricicoccus* genera, along with increased *Methanobrevibacter* and *Syntrophococcus* along with enteritis and inflammatory cells noted in the ileum (Xiang et al., 2019). Up to 16% of neonates afflicted with congenital parvovirus infection exhibit neurodevelopmental delay, which is likely a product of multiple synergistic mechanisms, including compromised fetal brain oxygenation from anemia, abnormal gut-brain development, and possibly direct neurological insult (Ornoy and Ergaz, 2017).

By the same token, CMV is a DNA virus of the herpesvirus family and a cause of congenital TORCH infection. In adults, CMV is well-known to cause enteritis, especially in immunocompromised hosts, those in shock states, those with concurrent pneumonia or chronic kidney disease, or with concurrent antibiotic exposure (Yeh et al., 2021). Does CMV have potential to inflict similar injury in the context of congenital infection? In the neonatal population, one study investigated neonatal intensive care unit admissions with patients infected with CMV at or close to birth, and found clinical signs ranging from abdominal distention, feeding intolerance, to diarrhea, protein-losing enteropathy, necrotizing enterocolitis, and associated hypernatremic hypovolemia (Cheong et al., 2004). Prenatally, congenital CMV may be detected on anatomy ultrasound with the presence of echogenic bowel, demonstrating that the early fetal gut may indeed be colonized or infected with these microbes well before birth (Nigro et al., 2012). Congenital CMV causes a robust innate immune response in the fetus, with monocytes, macrophages, and dendritic cells upregulating pro-inflammatory cytokines via toll-like receptors (TLRs) (Brizic et al., 2018). A recent study by Baasch et al. (2021) found that CMV infects macrophages and induces a stem-like state in these cells, impairs their antigen-presenting and phagocytic properties, and ramps up CMV replication in a mouse model of CMV. Mechanistically, they identified that these changes occur via upregulation of the transcription factor ZEB1 by Wnt signaling. It is possible that a similar process occurs in the context of embryonic/fetal CMV infection, although this remains to be shown.

We still lack evidence as to whether the cerebral effects of CMV (ventriculomegaly, sensorineural hearing loss, chorioretinitis, learning impairment) occur via direct insult from CMV particles entering the brain and causing an inflammatory response, or whether global disruptions in immune cells in the gut and circulation may also play a role, although we suspect that both are likely at play (Weisblum et al., 2014; Davis et al., 2017). Interestingly, polymorphisms in TNF-α and β-cytokine receptors can confer an increased risk of congential CMV infection, suggesting that some fetuses may be particularly susceptible to infection, and potentially explaining how many neonates are unaffected by maternal CMV infection (Arav-Boger et al., 2002). A future study to address this question could investigate whether neurocognitive disorders occur in neonates born with only CMV infecting the gut without any evidence of direct CNS involvement [for instance, infection occurring after formation of the blood brain barrier (BBB), or lacking the ultrasonographic hallmarks of periventricular plaques/calcifications].

One other member of the herpesvirus family and cause of congenital TORCH infection is herpes simplex virus (HSV), which upregulates inflammatory pathways and leads to neurological and gut inflammation. HSV enters fetal circulation through infection of Hofbauer cells, placental monocytes, and induces expression of IL-1β, a pro-inflammatory cytokine (Hendrix et al., 2020). The pro-inflammatory environment induced by HSV fosters multisystem organ dysfunction, as manifested by abnormal electroencephalogram (EEG) findings including seizure activity and potentially neonatal encephalopathy, as well as gastrointestinal/metabolic effects such as hepatic inflammation manifested by elevations in alanine aminotransferase (ALT) and aspartate aminotransferase (AST) with subsequent impaired glucose metabolism and hypoglycemia, correlating positively with degree of neonatal encephalopathy (O’Dea et al., 2020). From experimental studies in mice, it has been determined that HSV can infect the enteric nervous system, specifically the myenteric ganglia, leading to colonic dysmotility and toxic megacolon, a potential cause of severe neonatal morbidity and mortality in the context of this infection (Khoury-Hanold et al., 2016).

## Early Parasitic Infection Disrupting Gut-Brain Homeostasis

Aside from viruses, live pathogens also interfere with proper gut microbiome establishment with potential ramifications on proper CNS development. *Toxoplasma gondii* (*T. gondii*) is a cause of neonatal encephalitis and enteritis (Shao et al., 2020). In a mouse model of congenital toxoplasmosis, the cecal microbiome of mice infected with *T. gondii* is significantly altered, with increases in the relative abundance of Proteobacteria as well as harmful bacteria, such as *Bilopha* and *Desulfovibrio* species, and a decrease In *Lactobacillus* species (Shao et al., 2020). This bacterial dysbiosis appears to be mediated by macrophage induced nitrate production. Nitrate from activated macrophages serves as an energy source for enterobacter species (Wang et al., 2019). Mechanistically, infection with *T. gondii* causes intestinal dysbiosis by activating intestinal macrophages and upregulating nitrate production, leading to overgrowth of *Enterobacteriaceae* (Wang et al., 2019). Depletion of butyrate-producing organisms leads the colonic epithelium to increase expression of *Nos2* by surface epithelial cells via PPAR-γ signaling, thus increasing production of nitrate (Byndloss et al., 2017). Such changes in immune function could have far-reaching implications for proper brain homeostasis. For instance, microglia in the brains of *T. gondii*-infected fetuses cause focal nodules, likely through activation of complement signaling (Munday and Mason, 1979; Shinjyo et al., 2020). How the organisms gain access to the CNS is multifactorial: (1) BBB permeability is immature in the fetal brain, with tight junctions first expressed in the cerebral vasculature beginning at approximately 12 weeks of fetal life in humans and approximately E18 in mice; (2) chronic inflammation such as that caused by *T. gondii* infection, can promote increased BBB permeability and leukocyte infiltration through elevated interleukin-1β (IL-1β) signaling (Shaftel et al., 2007; Gov et al., 2013; Goasdoue et al., 2017; Kirschen et al., 2018). Thus, parasites may also gain access to the fetal brain and gut, leading to disruptive alterations in their maturation and creating an inflammatory environment with consequences on neurodevelopment.

## Intraamniotic Infection and Inflammation (III)

While some congenital infections may have a protracted, insidious course, affecting development across gestation, others present acutely with signs and symptoms of inflammation and a pronounced host response. One such example is III, also known as chorioamnionitis, an acute bacterial infection of the fetal membranes and placenta.

III is a common gestational complication associated with adverse infant outcomes such as preterm birth (PTB), neonatal sepsis, cardiopulmonary complications, and neurological injuries (Tita and Andrews, 2010). III can be defined as inflammation of the membranes and chorion of the placenta caused by the transmission of infectious organisms via retrograde infection from the lower genital tract (Tita and Andrews, 2010; Redline, 2012; Xiao et al., 2018). It can further be subdivided into three categories: histologic, microbiologic, and clinical. Histologic III is defined by pathological findings from placental histology such as funisitis or vasculitis; microbiological III involves the extraction of organisms from amniotic fluid or placental cultures; clinical III is characterized by maternal fever, maternal leukocytosis, and maternal and/or fetal tachycardia, which may be accompanied by uterine fundal tenderness or purulent vaginal discharge (Tita and Andrews, 2010; Xiao et al., 2018). III is responsible for a pro-inflammatory response by the fetus, known as Fetal Inflammatory Response Syndrome (FIRS), which can be characterized by an increase in the expression of the cytokines and chemokines IL-1, IL-6, CCL2, and TNF-α in the placenta and increased expression of IL-1β and TNF-α in the fetal brain (Tita and Andrews, 2010; Redline, 2012; Makinson et al., 2017; Hester et al., 2018; Stojanovska et al., 2018; Xiao et al., 2018; Chen et al., 2020; Briana et al., 2021). These biological markers are also indicative of alterations in neurological development.

Experimental models of III have utilized model organisms exposed to intrauterine lipopolysaccharide (LPS), allowing for the assessment of placental and fetal immune responses to a pro-inflammatory trigger (Redline, 2012; Makinson et al., 2017; Hester et al., 2018; Stojanovska et al., 2018; Xiao et al., 2018; Chen et al., 2020; Briana et al., 2021). In a study using mice, cytokines were measured 48 h after exposure to LPS. This study found that IL-1β, TNF-α, and CCL2 levels were increased in exposed mice, suggestive of recapitulation of human findings in III (Redline, 2012). Next looking at the brains of these pseudo-infected fetal mice, the investigators found white matter damage, increased numbers of microglia, and postnatal neurobehavioral changes, in particular with increased anxiety-related behaviors on elevated zero maze and light-dark box test, albeit with an anxiolytic-like phenotype on the open field test (Redline, 2012; Makinson et al., 2017). In another LPS-exposed mouse model, III significantly reduced hippocampal neurogenesis and granule cell density and resulted in improper ectopic granule cell migration (Hester et al., 2018). A separate experiment utilizing lambs exposed to LPS found reduced neuronal cell density, edema of the cortical gray matter, and blood vessel extravasation in the brain as a result of III (Stojanovska et al., 2018).

It is evident across a variety of studies that III has a significant impact on fetal brain development in ways that may be connected to alterations in the neonatal gut microbiome. In connection with long-term consequences, these physiological changes have been established as causes of neurological disorders such as cerebral palsy and autism and mental health conditions such as schizophrenia, anxiety, and depression (Makinson et al., 2017; Hester et al., 2018; Stojanovska et al., 2018; Chen et al., 2020; Briana and Malamitsi-Puchner, 2021). Changes to the fetal brain also harbor repercussions related to the fetal microbiome due to responses to III, with compromised placental or gut sterility, for example. Recently, studies have found that alterations to the fetal gut microbiome correlated with neurodevelopment and neuropsychiatric disorders in those previously afflicted with intrauterine infection (Ma et al., 2019; Warner, 2019).

A closer look at specific congenital infections resulting from III establishes the fetal response and adverse effects these infections can have on the fetal microbiome. For instance, Group B streptococcus (GBS) infection during labor or birth can lead to invasive GBS disease, encompassing a set of far-reaching sequelae on the neonatal organ systems, including alterations in neonatal intestinal composition, with reduction in *Bifidobacteria* or an increase of *Firmicutes* species (Aloisio et al., 2014; Stearns et al., 2017; Freudenhammer et al., 2021). As described in the next section, exposure to antibiotics to treat infections such as GBS may create their own set of issues related to disruption of the developing fetal gut, which may have complex effects on early development.

## Antibiotic Exposure on the Neonatal Gut Microbiome

Colonization of the gut is an essential step in the developing immune system. Initial gut microbiota is acquired during the neonatal phase via transmission of maternal microbes to the offspring both prenatally and during childbirth, marking it as a critical phase for sterility and immunity (Walker et al., 2017). External factors, such as antibiotics or dietary patterns, can affect the development of a healthy gut microbiome. Often to treat III and reduce the effects of congenital infections on both the mother and fetus, antibiotics are administered. A thorough investigation of the mechanisms and consequences antibiotics may have on the maternal and/or fetal gut microbiome is yet to occur, but there appears to be a clear trend in the studies that have been conducted thus far: early antibiotic exposure negatively influences the fetal gut microbiota, resulting in lasting effects on neurological development (Cantey et al., 2018; Eck et al., 2020; Zhou et al., 2020). Studies have found probiotics to have a positive outcome with regards to mitigating the harmful effects of antibiotic exposure (Rutten et al., 2015; Gong et al., 2021; Zhong et al., 2021).

**FIGURE 2:**
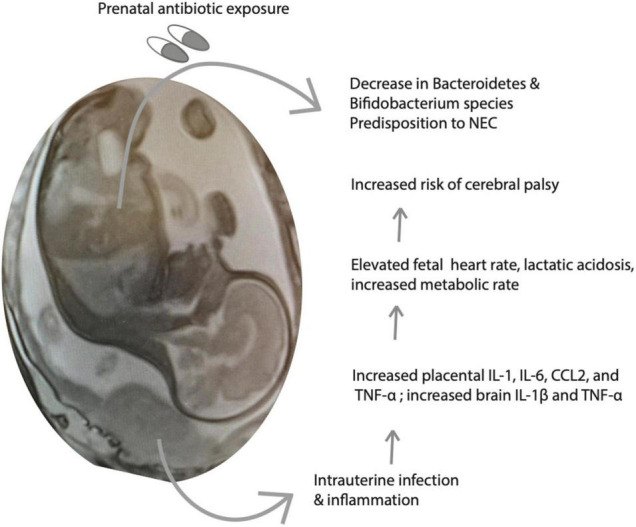
Intrauterine infection and inflammation (III) and treatment with antibiotics alter fetal metabolism. Shown is a schematic of the consequences of III on fetal inflammatory mediators and metabolic parameters, predisposing to increased risk of neurological injury and cerebral palsy. Antibiotics are important to treat the infection, however, they may have untoward consequences on neonatal gut flora maturation.

While antibiotics are viewed as essential to alleviating infection, their administration has been found to induce microbiota dysbiosis and create antibiotic resistant bacteria, resulting in increased fetal health risks. When mothers receive antibiotic treatment for infection, the vaginal microbiota that is passed to the offspring becomes unbalanced, which disrupts the development of the fetal microbiome (Zhou et al., 2020). One specific study analyzed *Lactobacillus* species from maternal vaginal swabs and neonatal meconium of mothers and neonates that did or did not receive antibiotics and found that groups who experienced antibiotic exposure had significantly lower loads of the bacteria (Zhou et al., 2020). The study suggested that exposed infants had elevated early onset sepsis due to this exposure as well (Zhou et al., 2020). In a different study investigating the development of intestinal microbiota post-antibiotic exposure in term infants, administration resulted in lower *Bacteroidetes* colonization and a differential development of the overall microbiome (Eck et al., 2020). A third study found that antibiotic use produced a less diverse microbiome, resulting in increased risks for early to late-onset sepsis, necrotizing enterocolitis (NEC), and in some cases, death (Cantey et al., 2018; Figure 2). NEC is the most common surgical emergency among preterm infants, and is more likely among infants weighing less than 1,500 g. Risk factors include prematurity, factors that predispose to gut dysbiosis, and lack of breastfeeding (Coggins et al., 2015). A metagenomic analysis of fecal samples performed by Olm et al. (2019) identified *Klebsiella* species, fimbriated bacteria, and bacteria that use quarom sensing as being significantly more common among NEC samples vs. control samples.

**FIGURE 3:**
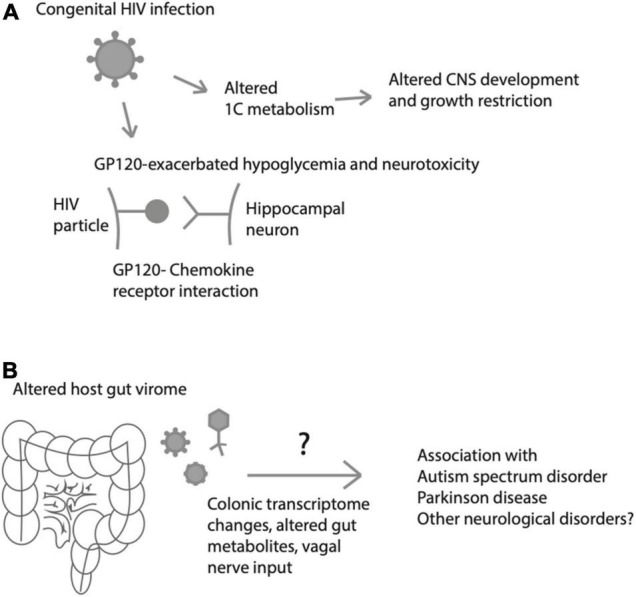
Effects of viruses on fetal/neonatal metabolism and neurological disease or injury. **(A)** Metabolic effects of HIV that may lead to altered fetal growth, central nervous system (CNS) development), glucose processing and excitotoxic brain injury. **(B)** Neonatal gut virome influences colonic transcriptome and gut metabolites, which may predispose to a variety of neurological disorders later in life.

In addition to antibiotics, antiviral medications may play some role in metabolic changes in the neonatal microbiome establishment. For instance, the infants of pregnant women infected with HIV have been found to have altered 1-carbon metabolism, potentially affecting the CNS (Surtees et al., 1990; Figure 3). It is unclear whether these changes are attributable to the virus *per se* or to highly active anti-retroviral therapy (HAART), well-known for causing adverse metabolic effects on lipid profiles, adiposity accumulation/redistribution, and cardiovascular disease (Levy et al., 2017). Although effects of HIV and/or HART on fetal development are generally considered minimal, they do include small for gestational age (SGA), low birth weight (LBW), and tenofovir may be associated with slower growth at 1 year of life (Jao et al., 2015). Additionally, experiments in rats have identified that hypoglycemic episodes (which are common in premature neonates) may be more common and may lead to exacerbated neurotoxicity in the hippocampus in the presence of the HIV protein GP120 acting on chemokine receptors (Barks et al., 1995; Figure 3).

It is evident that prenatal and postnatal antibiotic exposure affects the composition of the fetal and neonate gut microbiome, and further research has aimed to find a solution to diminish these effects. A promising potential remedy appears to be probiotic supplementation, as this can promote the growth of bacteria conducive to a healthy microbiome and restore the diversity of gut microbiota. In a randomized, controlled study, probiotics were administered simultaneously with antibiotics along with post-antibiotic treatment, resulting in the remodeling of the gut microbiota (Zhong et al., 2021). This suggests that probiotics may promote recovery or restoration of physiological neonatal gut microbiome formation following antibiotic treatment. Another study investigating term born infants exposed or not exposed to antibiotics focused on fecal samples obtained during the first year of life and found that the microbiota could be altered by probiotic administration to restore a healthy microbiome (Rutten et al., 2015). Similar work has been performed in preterm infants, with a smaller study investigating 40 premature infants finding that antibiotics increased pathogenic bacteria and reduced replication and repair functions as well as purine metabolism pathways (Gong et al., 2021). On the other hand, probiotic administration in these neonates increased the number of commensal bacteria, alleviating the negative effects of the antibiotics (Gong et al., 2021). Thus, while antibiotic exposure may lead to detrimental effects on the neonatal gut microbiome, further research is required to understand how probiotics or other early life interventions may be utilized to mitigate the effects of such antibiotics to protect the immunity of neonates.

Figure 2 depicts a summary of the interactions between perinatal infection and antibiotics, immune activation, and the developing gut-brain axis.

## Conclusion and Future Directions

Congenital infections have demonstrated profound effects on fetal and neonatal neurological development. The gut-brain axis has proven to be a pivotal participant in neurodevelopment, and congenital infections may contribute to abnormal maturation of the forming neonatal gut and developing brain. While the TORCH infections have been studied extensively, novel pathogens including COVID-19 and ZIKV will require further investigation to understand their full impact on the developing gut-brain axis. By the same token, antibiotics are often used prenatally and in the early postnatal period for treatment of conditions such as intraamniotic infection, maternal pulmonary or urinary tract infections, or neonatal bacterial infections. These treatments are often employed without consideration of potential effects on the developing gut, yet, this early exposure to antibiotics likely affects the developing gut microbiome in ways that will impact gastrointestinal and systemic health for the lifespan of the individual.

We are beginning to understand that the CNS communicates with systemic circulation likely via microglia, the brain immune cells that sense blood-borne cues that may be gut-derived to influence activation and inflammatory responses. We look forward to future investigations of the relationship between the developing gut-brain axis and early life exposures to pathogens and antibiotics, which may hold the key to still incompletely understood diseases such as schizophrenia, mood disorders, and cerebral palsy.

Here, we have focused largely on the neonatal gut microbiome, with a bias toward bacterial organisms. However, this discussion neglects an increasingly recognized large number of viruses that colonize the neonatal gut and may have important roles in metabolic homeostasis and gut maturation. While under physiological circumstances, the gut is entirely devoid of viruses at birth, recent work from Liang et al. (2020) has shown that following bacterial colonization of the neonatal gut, bacteriophages come to inhabit the gut by 1–4 months of life, as measured by quantitative polymerase chain reaction (qPCR) of meconium and stool samples of neonates. Importantly, whether the infant is breastfed vs. formula-fed influences the bacterial as well as the viral landscapes of the developing infant gut, suggesting a role of immune activity (either from passive immunity from breastmilk-derived antibodies or from maternal skin flora passing into the fetal gut) on both viral and bacterial colonization of the developing gut (Liang et al., 2020). The gut virome is also an increasingly recognized contributor to proper neurological health, with altered host virome-brain interactions being implicated in disorders including autism spectrum disorder and Parkinson disease, perhaps through perturbations in the colonic transcriptome (Kang et al., 2017; Mihindukulasuriya et al., 2021; Stockdale et al., 2021; Figure 3). How these interactions between immune system, gut microbiome and virome and neurological systems interact in normal and pathological ways remains an open question that will require more basic and clinical investigation.

## Author Contributions

GK and SP wrote the original draft. IB edited the draft. All authors proofread and edited the final draft and agreed with the final version.

## Conflict of Interest

The authors declare that the research was conducted in the absence of any commercial or financial relationships that could be construed as a potential conflict of interest.

## Publisher’s Note

All claims expressed in this article are solely those of the authors and do not necessarily represent those of their affiliated organizations, or those of the publisher, the editors and the reviewers. Any product that may be evaluated in this article, or claim that may be made by its manufacturer, is not guaranteed or endorsed by the publisher.
